# Feasibility and preliminary effects of a culturally adapted short message service intervention for non-communicable disease prevention in older adults: a pilot study

**DOI:** 10.7717/peerj.21643

**Published:** 2026-08-24

**Authors:** Nour Amin Elsahoryi, Fadia Milhem

**Affiliations:** Department of Nutrition, Faculty of Pharmacy and Medical Sciences, University of Petra, Amman, Jordan

**Keywords:** Pilot study, Feasibility study, Noncommunicable diseases (NCDs), Older adults, Mobile health (mHealth), Text messaging intervention, Knowledge, Attitudes, and Practices (KAP)

## Abstract

**Background:**

Among low- and middle-income countries, noncommunicable diseases (NCDs) are a significant health burden among the elderly. Mobile health interventions provide resource-constrained health education that is scalable.

**Aims:**

Determine the viability, acceptability, and preliminary effects of culturally adapted Short Message Service (SMS)-based nutrition and lifestyle change on the knowledge, attitudes, and practices (KAP) of older adults in Jordan towards prevention of noncommunicable diseases (NCDs).

**Method and Materials:**

A total of 79 older adults (mean age 57.33 ± 9.37 years) with NCDs received 30 culturally tailored SMS messages daily for one month. Messages were based on Social Cognitive Theory and addressed dietary habits, physical activity, and NCD risk reduction. A validated Arabic-adapted KAP questionnaire was administered pre- and post-intervention.

**Statistical Analysis:**

Paired-samples *t*-tests were used to compare pre- and post-KAPs. Effect sizes were calculated using Cohen’s *d*. Sociodemographic factors associated with KAP improvement were examined through multiple linear regression (*p* ≤ 0.05).

**Results:**

Significant improvements occurred in knowledge (mean difference = 40.62, *p* ≤ 0.001; Cohen’s *d* = 3.29) and attitudes (mean difference = 24.43, *p* ≤ 0.001; Cohen’s *d* = 1.77). There was no significant change in practice scores (*p* = 0.175). Overall KAP improved significantly (*p* ≤ 0.001; Cohen’s *d* = 2.15), driven primarily by gains in knowledge and attitudes; practice scores did not change significantly. Greater improvement in KAP was associated with higher education (*β* = 0.379, *p* ≤ 0.001), younger age (*β* =  − 0.646, *p* ≤ 0.001), female sex (*β* =  − 0.067, *p* = 0.007), and living alone (*β* = 0.150, *p* = 0.031).

**Conclusions:**

The pilot study indicates that culturally adapted SMS messaging among older adults with NCDs is feasible and acceptable in Jordan, and its early impacts on knowledge and attitudes are promising. These results provide preliminary evidence and effect size estimates needed to design a fully powered randomized controlled trial to determine effectiveness.

## Introduction

The term non-communicable diseases (NCDs) refers to diseases that are usually persistent and chronic and may be caused by a combination of factors including genetic, hereditary, physiological, environmental, and behavioral factors. These diseases are divided into several non-communicable diseases, which are cardiovascular diseases, respiratory diseases, diabetes and cancers (Yin et al., 2024). According to global studies and statistics, the rate of NCDs is increasing, which poses a major challenge to health systems in terms of the cost of treatment and the economic burden on the state and society, where NCDs account for 74% of deaths worldwide (WHO, 2021). Older adults represent a population disproportionately affected by non-communicable diseases and their complications, so educational and awareness interventions about these diseases are crucial for these patients, which may reduce the cost of treatment for the patient, the increase in symptoms, and the development of complications, and improve the patient’s quality of life (Vos et al., 2020). Globally, between 2011 and 2030, the estimated cost of the NCD diseases exceeds 30 trillion US dollars, and 48% of the global GBD (GBD 2019 Diseases and Injuries Collaborators, 2024). In developing countries, the economic level is not high, and medical resources are limited.

The delayed provision of evidence-based therapeutic interventions represents a critical barrier to optimal patient outcomes, contributing to suboptimal disease management and consequently elevating mortality rates and functional disability among affected populations (Vos et al., 2020). The complex nature of non-communicable diseases necessitates sustained clinical engagement encompassing long-term therapeutic protocols and comprehensive disease management strategies, making patient education programs that target lifestyle modification particularly crucial for therapeutic success (UN, 2018). These organized educational programs are proven to be effective in preventing negative clinical outcomes and decreasing the general burden of the disease by promoting evidence-based lifestyle changes and behavioral adjustments (Benjamin et al., 2019). The challenges of achieving the best patient results and managing risk factors depend on multidimensional strategies that combine patient empowerment through lifestyle education, environmental health governance, and strong implementation of public health policy (Woodward et al., 2024). The highly relevant role of increasing patient awareness of the modifiable risk factors of non-communicable diseases and providing systemic advice on the evidence-based modifications in lifestyle, which can fulfill the dual functions of primary prevention processes and secondary interventions to prevent the development of complications and prevent further progress of symptoms, is supported by current health promotion practices (Jamison et al., 2006; Yin et al., 2024). The digital health education concept has emerged as a promising alternative in offering patients with non-communicable diseases evidence-based health education through internet-based learning environments that can be utilized to enhance patient self-direction in managing their diseases (Matheson et al., 2013). The implementation of technology-mediated health communication methods among the older adult population is a social requirement and a clinical need because increasing numbers of health complications and care requirements associated with age are becoming common among the elderly (Mace, Mattos & Vranceanu, 2022). New avenues of delivery of health education made available by the epidemic utilization of internet enabled mobile devices have provided previously unavailable opportunities in the delivery of highly targeted health education to the elderly population that promises to transform the lifestyle and health benefits thereof to a significant degree (Jamison et al., 2006; Whitehead & Seaton, 2016). There are some indicators that technology-based educational interventions may be effective in changing health-related behaviors as well as reducing harmful lifestyle practices, particularly those concerning dietary habits and exercise patterns, which are the main modifiable risk factors in the etiology of non-communicable disease complications and the worsening of symptoms (Shiferaw, 2020; Creighton et al., 2025). Remote health education provided on the platform of the latest and most technologically advanced technological solutions has certain advantages to the aging population that frequently experiences mobility disorders and transportation issues, and the availability of the entire spectrum of universal and specialized health care is expanded to the population at risk (Anawade, Sharma & Gahane, 2024; Yin et al., 2024). All e-learning programs include online courses, newsletters, social media messages and successful nutritional counseling. Such programmes can promote healthy living, managing food quantities, choosing the right type and quantity of food, managing the volume of sugar and salt in the diet, and changing exercise habits to promote healthy lifestyles in older adults (Chau, Burgermaster & Mamykina, 2018; Ayaz-Alkaya et al., 2020). Some research has been conducted on the impact of educational intervention in such programs on healthy lifestyles of non-communicable disease patients, however, there is no agreement on the impact of such interventions on the life of the patient (Yin et al., 2024). Conversely, there are very limited studies that illuminate the awareness of the elderly and even the limited ones that concentrate on chronic and NCDs. Moreover, the studies that have been done so far are cross-sectional studies, not education interventions. For example; Williamson et al. (2021) evaluated the effect of patient education and exercise sessions on the knowledge of 90 patients with coronary artery disease. The results showed a significant improvement in knowledge of the disease and a greater awareness of the importance of rehabilitation after the educational sessions (Williamson et al., 2021). However, this study included educational sessions in the form of face-to-face groups rather than electronically, and there was poor commitment to attendance among patients (Williamson et al., 2021).

Short Message Service (SMS) based interventions in non-Arab Middle Eastern settings have also shown some degree of success; for example, a study on SMS education in Iran for patients with type 2 diabetes showed that SMS educational interventions over a period of several months had a measurable impact on self-efficacy and self-management behaviors, suggesting the SMS modality is useful outside the specific cultural context of the Arab world in which the present study is set (Goodarzi et al., 2012). Likewise, a large meta-analysis of text messaging interventions conducted in Western and high-income contexts showed similar positive impact on health knowledge and health behaviors across several chronic disease contexts, but noted that the relevance of content and the cultural fit of programs were important moderators of effectiveness, emphasizing the need to adapt programs to the local context before achieving a health change (Head et al., 2013). Conversely, a pretest-posttest study that enrolled 30 aged women was intended to inform the effect of educative programs on their lifestyle choices and general health awareness. The means of implementation of the intervention was home visits because it was the major source of education and raising awareness. Nonetheless, the results revealed that the level of health awareness of the participants did not change, as a statistically significant result, after the intervention (Ayaz-Alkaya et al., 2020). As for cross-sectional studies, Koohi & Khalili (2020) reported that the general knowledge and attitude regarding cardiocvasculare diseases was high and satisfied but the nutrition, smoking and physical activity behaviors was not satisfied among Iraninan adults attending (HCC) Health Care Centers in Tehran. Another cross-sectional study among Lebanese population reported limited knowledge, attitude, and practice (KAP) toward CVDs (Machaalani et al., 2022). Similarly, good knowledge and attitude regarding CVD risk factors were reported by convenient sample in Malaysia (Mohammad, Rahman & Haque, 2018). Moreover, Yenit et al. (2023) study reported that self-efficacy among health workers (HEWs) in Ethiopia was a factor in raising knowledge of NCDs and improving physical activity, and that there was a need to include healthy lifestyle in health worker training. In contrast, (Ojo et al., 2017) reported a deficiency in the knowledge regards NCDs in Eastern Uganda communities, and they concluded that the health awareness teams can play an effective role in preventing and improving the NCDs management through local and national educational initiatives. Similar findings in a study conducted on urban respondents indicated that KAP regarding NCDs is insufficient and there is a need to understand the factors that led to these results in the urban community (Ithnin et al., 2021). Nevertheless, the viability of SMS-based interventions in preventing NCD among Arab older adults is not studied in pilots, and no evidence is available to understand whether SMS-based interventions are acceptable and implementable within primary care services in Jordan. To address this gap, the present study adopted Social Cognitive Theory as its theoretical foundation, given its established utility in explaining how knowledge development, self-efficacy beliefs, and anticipated outcomes jointly drive health-related behavior change (Islam et al., 2023).

As far as we are aware, none of the prior studies in the Arab world have designed and tested a set of culturally appropriate educational messages in Arabic language to enhance nutritional and lifestyle behaviors among older adults with NCDs. Also, no NCD-related KAP tool that is validated in Arabic language exists. Cardiovascular disease was chosen as the focus for this intervention as it is the leading cause of NCD-related mortality in Jordan—national surveillance data indicate that approximately 24.5% of Jordanians aged 40–69 years carry a 10-year CVD risk of ≥30% or have existing CVD, underscoring the particular urgency of targeted prevention efforts in this context (Ministry of Health, Hashemite Kingdom of Jordan, 2020). Therefore, the proposed pilot pre-post intervention research will achieve the following objectives: (1) determine the feasibility and acceptability of introducing daily SMS messages to older adults with NCDs in Jordanian primary care facilities; (2) address the primary preliminary outcomes in terms of knowledge, attitudes, and practices (KAP) concerning nutrition and physical activity; and (3) offer an Arabic-language validated instrument in the evaluation of NCD-related KAP, which will guide the development of a further randomized controlled trial. This study focuses on the potential of targeted, accessible education messages to help create more effective and scalable interventions to prevent NCDs.

## Material and Methods

### Study design

In this pilot study, a one-group pre-post design was used to determine the viability of administering SMS messages to older adults with NCDs and to get preliminary estimates of the effect on knowledge, attitudes, and practices (KAP) related to cardiovascular disease prevention. The pilot design was selected to guide the calculations of the sample size and intervention revisions of a future definitive randomized controlled trial. The program entailed text messages daily over 4 weeks with a specific purpose of increasing health awareness, strengthening good health attitudes and encouraging the adoption of health behavior that is helpful to health. The study design follows the precedence established by earlier studies that have validated the effectiveness of before-and-after analysis models to evaluate behavioral and educational change particularly in population groups of the elderly (Ayaz-Alkaya et al., 2020; Williamson et al., 2021). Health interventions through the use of text messages have been demonstrated as cost-effective and readily available means of health education delivery and promoting behavioral change in older populations and groups with limited access to routine medical services (Head et al., 2013; Dobson et al., 2024).

The theoretical basis of the intervention is described in the Introduction. The study was conducted with written consent of the participants, and the study protocol was approved by the University of Petra Research Ethics Committee (Reference: E/H/4/10/2024) with the assurance of adherence to the principles of confidentiality and voluntary participation. The before-and-after method was selected to be an appropriate method of measuring individual level improvements in KAP after exposure to this targeted, time-limited intervention. The 30 day period was selected to be optimal for a pilot feasibility study in which the main objective was not a demonstration of conclusive behavioral changes (which are known to take 3–6 months to result) but to determine the logistical sustainability of the intervention, participant adherence, and initial estimates of effect size to inform a future definitive trial (Thabane et al., 2010).

### Feasibility and acceptability outcomes

Primary outcomes were pre-defined measures of feasibility and acceptability, as would be in a pilot study. The recruitment rate (percentage of those eligible to participate who did so) and the retention rate (percentage of those who enrolled who completed follow-up assessment) were used to operationalize feasibility, with a recruitment rate of ≥70% and a retention rate of ≥70% being considered as indicative of feasibility. Self-reported intervention adherence (message reception and readability) and participant satisfaction collected with structured feedback in follow-up interviews were used to assess acceptability, with ≥70% of participants defining acceptable uptake as a positive response (Bowen et al., 2009).

Preliminary effect estimates of the changes in KAP scores were the secondary outcomes that would be used to determine the power in future investigations.

### Participants

A convenience sample of older adults was recruited from four governmental primary healthcare centers located in different districts of Amman, Jordan. Recruitment was conducted between April and June 2024 through in-person invitations by trained healthcare staff during routine clinic visits. Participants were approached individually and provided with detailed information about the study prior to consent. The inclusion criteria were: age 50 years and over; confirmed clinical diagnosis of at least one non-communicable disease (NCD) (*e.g.*, cardiovascular disease, type 2 diabetes, hypertension); residing in the catchment area of the selected NCD centers; being able to read and understand Arabic; and having the ability to receive and read SMS messages without assistance from anybody. The following were excluded: (1) documented and/or suspected cognitive impairment that interfered with informed consent or study participation; (2) severe visual or hearing limitations that prevented engagement with the SMS content; (3) having a medical diagnosis of any acute or terminal illness that required hospitalization or palliative care (*e.g.*, pneumonia, metastatic cancer); or (4) already participating in another structured health education program during the study period. A brief structured screening checklist was administered by health center staff before participation to ensure that participants were eligible and to reduce bias. The anonymity of all the participants was respected, and they signed informed consent forms; the data were treated with complete confidentiality and in accordance with the institutional ethical guidelines.

### Sample size

To calculate the minimum sample size needed to be analyzed by a paired *t*-test to compare pre- and post-intervention outcomes, a priori power analysis was performed using G*Power version 3.1.9.7. A medium effect size (Cohen’s *d* = 0.5), one-tailed significance level of 0.05 and a desired statistical power of 95% resulted in a required total sample size of 45 participants. A greater number of 100 older adults were recruited at the outset in order to make the sample size larger and have a larger number of participants for analysis, while being aware that some participants might drop out. During the study period, 21 participants either withdrew voluntarily, were lost to follow-up, or failed to complete the post-intervention assessment. Consequently, the final analytical sample comprised 79 participants who completed both the baseline and follow-up evaluations, thereby exceeding the minimum required sample size of 45 participants.

The Cohen’s *d* values obtained from this single-arm pilot should be viewed in the context of this convenience sample. These effect sizes are shown with 95% confidence intervals for the true mean difference (shown in the Results section) and are not intended to be used as definitive measures of effect but rather as initial estimates to determine if the effect is confirmed in a future randomized trial. Participants who withdrew or did not complete a follow-up assessment were removed from the final dataset, and the latter was analyzed using a per protocol approach, with analyses performed only in individuals who received their full complement of baseline and post-intervention tests (*n* = 79).

### Intervention procedure

Participants were interviewed face-to-face by trained members of the research team affiliated with the Department of Clinical Nutrition and Dietetics at University of Petra. These interviews were conducted in the waiting areas of the participating health centers before the start of the study. During the interview, each participant received a clear explanation about the study objectives, the nature of the intervention, the type and timing of the SMS messages, and the requirement to complete questionnaires before and after the intervention. After confirming eligibility, participants provided written informed consent and completed the baseline questionnaire under the direct supervision and guidance of a trained dietitian, who offered clarifications when needed. Interviews were circa 10 to 20 min. The intervention was a combination of one standardized daily health- promoting Short Message Service (SMS) message (identical, cost-effective and technology-based intervention) delivered for 30 consecutive days. At 10:00 a.m., all messages were sent, a time that was pre-approved by all participants as being suitable and convenient for reading all the messages (although messages read after 10:00 a.m. were allowed). The messages were all sent to participants in one direction only by one member of the research team on a common institutional mobile telephone, thus ensuring uniformity of exposure to the 30 messages. All the messages focused on cardiovascular disease prevention, as well as on general healthy lifestyle and dietary behavior change. The messages were based on evidence-informed content and were clear, culturally appropriate, and actionable. Through this standardized method of sending messages, we ensured stable exposure, optimized interactions and reduced differences in message reception and decoding among the participants.

### Mobile messages

In this intervention, educational SMS messages were developed through a rigorous and structured process of development, in line with international cardiovascular prevention guidelines and proven behavioral change theories. The model used to develop message was adopted from the study conducted by Redfern et al. (2014) that was used for development of the prompts in the processing of the promotional messages for smoking cessation, dieting and physical activity in order to achieve a healthy lifestyle to reduce cardiovascular risk (Redfern et al., 2014).

In this study, the research team reviewed and selected 30 core messages from the Redfern framework (Redfern et al., 2014) and made significant modifications and cultural adaptation of these messages, to make them suitable for the older adult in Jordan, who speaks Arabic and has certain beliefs about health which shape their behavior. English messages were not merely translated from other languages but instead were reformulated by people who are experts on public health, nutrition research, and clinical nutrition with backgrounds in chronic disease prevention. It ensured linguistic clarity, cultural relevance, and was based upon local dietary traditions and public health issues. The adapted messages were reviewed by a panel of three bilingual public health and clinical nutrition experts, and pre-tested with a small convenience sample of five older adults with NCDs not enrolled in the main study to confirm appropriateness of the adapted messages. The wording and comprehensibility were improved with feedback. Regarding the length of the messages, all messages were selected to fit within the 160 characters limit of a single SMS and were also tested to ensure readability by older people with different levels of health literacy, following the recommendations of digital health interventions targeting older people (Mace, Mattos & Vranceanu, 2022). Examples of the types of messages include: ‘Walking for 30 min a day keeps your heart healthy and helps you lower your blood pressure but try starting with 10 min after breakfast’ and ‘Fill half your plate with vegetables at every meal—your heart will thank you. The text was written in simple, everyday Arabic, was free from medical jargon and contained only one actionable recommendation per message to reduce the cognitive load for older readers.

The messages designed based on the behavior changes techniques, included increasing knowledge, support the self-efficacy, encouraging self-mentoring, reinforcing motivation, reduce the effect of risk situation, and prevents the lapse (Redfern et al., 2014),

In addition, the messages were formulated in a simple, encouraging, and positive manner, free of any negative or punitive phrases. The messages focused on adopting a healthy lifestyle, including physical activity such as daily walking, improving dietary patterns such as increasing dietary fiber, educating patients about healthy foods and encouraging them to eat them, drinking plenty of water, and other health considerations specific to them, in accordance with international recommendations. The messages included quantitative and qualitative descriptions of food types, such as the recommended daily portions of fruits and vegetables, and the types and quantities of oils commonly used in Jordan as part of the Middle East. In addition, the messages included recommendations and awareness about dietary habits, such as the number of meals and reading food labels, making these messages easily applicable to older adults with NCDs.

Once daily, a unidirectional messaging format was used for all messages, delivered by SMS (short message service) at 10:00 a.m., a time chosen by participant consensus during enrollment as the most optimal for receiving and reading the messages. The information was identical and all participants received the same messages at the same time. A standardized approach was necessary to maintain the validity of internal processes and to evaluate the group level effect of the intervention. Taking the evidence-based guidelines, behavioral science, and cultural tailoring together, the final SMS set intended to instigate heart-healthy behaviors and enhance awareness and lifestyle practice for older adults with NCDs in Jordan.

### Study questionnaire

In this study, the (KAP) questionnaire used was a modified Arabic adaptation of the validated CVD-KAP29 tool (Koohi & Khalili, 2020; Koohi et al., 2021), which was originally developed and psychometrically tested for evaluating the role of KAP with respect to cardiovascular disease among adults. The validity and reliability indicators of the original questionnaire were robust: they demonstrated the construct validity as they were confirmed by the exploratory and confirmatory factor analysis and the internal consistency indices (Cronbach’s *α* > 0.60). The questionnaire in the present study was historically and contextually adapted to suit the Jordanian older adult population, plus the wider realm of (NCDs) and not only cardiovascular disease. The language was improved linguistically in Arabic through refining the terminology and culturally sensitive or irrelevant items were left out (*e.g.*, alcohol related questions) and content was adapted to include the main NCDs in Jordan such as; diabetes, hypertension and obesity.

The final instrument comprised of 29 items divided into three domains: Knowledge domain (12 items): Participants were tested on their understanding of the knowledge of the NCDs, their risk factors (*e.g.*, smoking, unhealthy diet, physical inactivity), their symptoms and complications. The answers were graded on a three-point scale (2 = Yes, 1 = I don’t know, 0 = No) and the grades were higher for better knowledge. Attitude domain (10 items): beliefs, perceptions and motivation about engaging in preventive behaviors. The answers were captured on a five-point Likert scale (1 = Strongly Disagree to 5 = Strongly Agree). Practice domain (seven items): assessed participants’ involvement in healthy behaviors (physical activity, diet pattern, smoking cessation, and stress management). The items were answered with binary scoring (1 = yes, 0 = no), with the exception of the items related to the sources of fat and salt use in food, where a categorical response was required. Personal sociodemographic data (age, gender, education, marital status, chronic disease history, and smoking status) were also collected. The questionnaire was self-administered in two phases: baseline (pre-intervention) and post-intervention (after receiving 30 text messages over one month). During both time points, trained data collectors assisted participants in completing the questionnaire through structured interviews conducted at health centers. Instructions were standardized to ensure consistency and minimize interviewer bias. Content validity of the adapted tool was re-reviewed by a panel of nutritionists and public health experts and pretested in a small pilot to confirm comprehension and cultural appropriateness prior to full deployment. To interpret KAP scores, we used the categorization framework proposed in the original study: total scores were classified as “Very Insufficient” (≤20), “Insufficient” (21–40), “Sufficient” (41–60), “Satisfactory” (61–80), and “Very Satisfactory” (>80), allowing for both individual-level and group-level comparison of KAP shifts before and after the intervention (Koohi & Khalili, 2020).

### Psychometric analysis of the Arabic-modified questionnaire

No Arabic translated version of this questionnaire has been previously validated, therefore, the Arabic modified questionnaire was evaluated with respect to its validity and reliability in the present study sample (*n* = 79). This method was needed because the tool had not been used before in the context it was adapted for, but is recognized as a limitation, and the need to validate it in a separate sample before widespread use of the instrument is recommended.

Validity was established through several methods. Face validity was ensured by refining the Arabic translation to ensure clarity and cultural relevance. This was done by consulting with bilingual experts in health and epidemiology (Field, 2018). Content validity was confirmed by expert reviews, where a panel of professionals in cardiovascular health, epidemiology, and nutrition examined the items to ensure comprehensive coverage of the key factors related to CVD KAP (Tabachnick & Fidell, 2019). Construct validity was assessed using Exploratory Factor Analysis (EFA) with principal axis factoring and varimax rotation (Hair et al., 2022). The Kaiser-Meyer-Olkin (KMO) test (0.81) indicated an acceptable sampling adequacy and Bartlett’s test of sphericity (*χ*^2^(120) = 985.47, *p* ≤ 0.001) supported the appropriateness of factor analysis. Pearson correlations between the scores on the questionnaire and the participants’ behaviors associated with cardiovascular health showed moderate to strong correlations (Field, 2018). The results indicated that the questionnaire had a comprehensiveness to include the appropriate constructs of CVD KAP. Cronbach’s alpha was used to test the internal consistency of the instrument for reliability. The results showed that there was a moderate internal consensus (*α* = 0.68), that is the internal consistency is acceptable but can be improved (Hair et al., 2022). A randomly selected sub-sample of 50 participants participated in test–retest reliability assessments, filling out the questionnaire at two time points with a two-week interval between the administrations, under the same administration conditions as in the main study (structured interview format at the health centers). Pearson correlation values between the two administration ranged from 0.54–0.72 (*p* ≤ 0.001) indicating moderate to high temporal stability. The inter-rater reliability was measured by intraclass correlation coefficient (ICC) and found to be 0.79, which is a good agreement among the raters (Field, 2018). All data were analyzed using SPSS version 25. The overall reliability of this questionnaire is moderate, but improvements are needed to achieve an increased level of internal consistency. The four-factor structure identified through EFA accounted for 62.4% of the total variance, consistent with the theoretical model underlying the instrument (Hair et al., 2022).

### Statistical analysis

Data were analyzed using IBM SPSS Statistics for Windows 25.0 (IBM Corp., Armonk, NY, USA). Overall KAP score change (post intervention–pre intervention) was the dependent variable. The changes in individual KAP domain scores (knowledge, attitudes, and practices) were the secondary dependent variables. Independent variables included were sociodemographic (age, gender, education, marital status, employment status, and living status) and lifestyle-related (BMI, smoking status, and physical activity status). The sociodemographic data of the participants were summarized using descriptive statistics. Normality of continuous variables (age and BMI) checked by Shapiro–Wilk test (Ghasemi & Zahediasl, 2012), and since they followed a normal distribution, they were reported as means ± standard deviations (SD). BMI was calculated as weight in kilograms divided by height in meters squared (kg/m^2^), measured by trained staff using standardized equipment at the time of enrollment. Categorical variables were summarized as frequencies and percentages (%). A paired-samples *t*-test was used to compare KAP scores before and after the education intervention. Results were reported as means ± SD, with mean differences, 95% confidence intervals (CIs), and *p*-values. Cohen’s *d* measured effect size, interpreted as small (≥0.2), moderate (≥0.5), or large (≥0.8). KAP scores were converted into categories for clearer analysis into five levels based on the original article (Koohi & Khalili, 2020; Koohi et al., 2021): “Highly Insufficient” (≤20), “Insufficient” (21–40), “Sufficient” (41–60), “Satisfactory” (61–80), and “Highly Satisfactory” (>80). A multiple linear regression analysis was conducted to examine the relationship between sociodemographic factors and changes in KAP scores (KAP difference). The dependent variable was KAP difference, while independent variables included age (continuous), BMI (continuous), gender (categorical), education level (categorical), employment status (categorical), living status (categorical), smoking status (categorical), and physical activity level (categorical). Prior to analysis, the normality of continuous variables (age and BMI) was assessed and confirmed, ensuring the appropriateness of parametric testing. All independent variables were entered at the same time in the linear regression analysis (Enter method). Listwise deletion was performed to ensure data integrity when dealing with missing data. Normality of residuals, linearity of the model, homoscedasticity and multicollinearity were checked. Standardized and unstandardized residuals were saved and checked for potential outliers with corrective action taken as needed. The Durbin-Watson statistics were also computed to check for the presence of autocorrelation. All coefficients were tested for statistical significance at *p* < 0.05 and reported along with 95% confidence intervals to assure sound inferences about the data.

## Results

### Feasibility and acceptability outcomes

There were good indicators of feasibility of the intervention. Out of 120 older adults who were approached, 100 of them agreed to participate (recruitment rate: 83.3%). Out of them, 79 participants filled out the baseline and follow-up assessment (retention rate: 79%). While it is known that SMS delivery across networks can vary across the region, participants, in follow-up interviews, indicated that they received all or almost all of the messages daily, with a small minority reporting some instances of non-delivery; informal responses indicated that participants were very happy with the timing of messages (10:00 AM); they also indicated a high level of satisfaction with the languages and cultural appropriateness of the messages.

### Demographic and health characteristics of the study participants

The study included 79 participants with a mean age of 57.33 ± 9.37 years and a mean body mass index (BMI) of 28.23 ± 4.87 kg/m^2^. Females constituted 87.3% of the participants, while males constituted 12.7%. The majority were married (72.2%), while 10.1% were single, and 17.7% were divorced or widowed. Regarding educational level, 50.6% had a high school diploma or lower, 26.6% had a bachelor’s degree or diploma, and 22.8% had a postgraduate degree. Regarding employment status, 46.8% were employed, 30.4% were unemployed, and 22.8% were retired. The majority of participants (49.4%) lived alone and 50.6% lived with their families. As for physical activity, 57.0% of them didn’t participate in any physical activity, 32.9% occasionally participated in physical activity, and 10.1% participated in physical activity regularly. Most prevalent health conditions included high blood pressure (49.4%), diabetes (29.1%), heart and kidney diseases (6.3%) and other diseases (15.2%). Regarding smoking, 29.1 percent were non-smokers, 6.3 percent were former smokers, and 64.6 percent were current smokers. The demographic and health characteristics of the study participants are presented in Table 1.

**Table 1:** Demographic, lifestyle, and clinical characteristics of older adults with NCDs included in the SMS intervention. Values are presented as mean ± SD for continuous variables and *n* (%) for categorical variables. Chronic disease categories represent the primary clinical diagnosis recorded in health-center records; each participant was classified into one disease category. BMI, body mass index; NCDs, noncommunicable diseases; SMS, short message service.

Characteristic	Categories	*N*(%)
Age	57.33 ± 9.37	
BMI	28.23 ± 4.87	
Gender	Male	10 (12.7%)
	Female	69 (87.3%)
Marital status	Single	8 (10.1%)
	Married	57 (72.2%)
	Divorced/Widowed	14 (17.7%)
Education level	High school or less	40 (50.6%)
	Bachelor’s/Diploma	21 (26.6%)
	Postgraduate	18 (22.8%)
Work status	Retired	18 (22.8%)
	Unemployed	24 (30.4%)
	Employed	37 (46.8%)
Living status	Living alone	39 (49.4%)
	Living with family	40 (50.6%)
Physical activity	Not exercising	45 (57.0%)
	Sometimes	26 (32.9%)
	Regularly	8 (10.1%)
Chronic disease^a^	Hypertension	39 (49.4%)
	Diabetes	23 (29.1%)
	Heart/Kidney disease	5 (6.3%)
	Other	12 (15.2%)
Smoking status	Non-smoker	23 (29.1%)
	Former smoker	5 (6.3%)
	Current smoker	51 (64.6%)

**Notes.**

a Chronic disease categories reflect each participant’s primary clinical diagnosis as recorded in health center records; participants were classified into a single disease category, and frequencies therefore sum to the total sample (*n* = 79).

### Pre-post changes in knowledge, attitude, and practice

**Figure 1:**
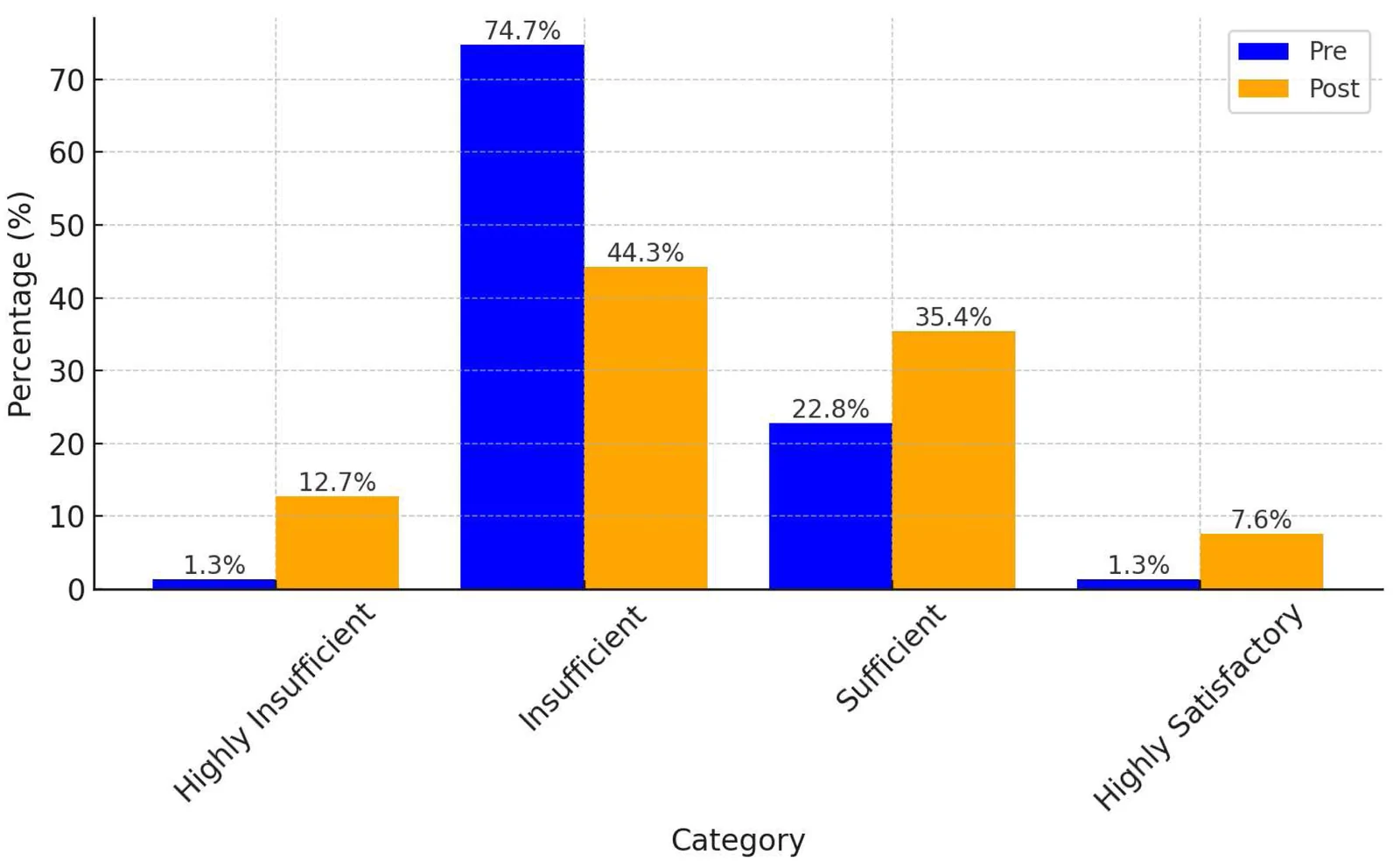
Distribution of overall KAP categories before and after the SMS intervention among older adults with NCDs. The percentage distribution of participants across overall KAP categories before and after the 30-day SMS-based educational intervention. The post-intervention distribution shifted from predominantly insufficient scores toward sufficient and highly satisfactory categories, indicating improved overall KAP classification after the intervention. KAP, knowledge, attitude, and practice; NCDs, noncommunicable diseases; SMS, short message service.

Table 2 data indicated that there was a significant increase in the knowledge and attitude components after the intervention, and a non-significant positive trend in the practice components. Knowledge demonstrated the greatest change, with a mean increase of 40.62 points and a large effect size (Cohen’s *d* = 3.29, *p* ≤ 0.001). Attitude also showed a great mean difference and effect size (Cohen’s *d* = 1.77, *p* ≤ 0.001). A small, non-significant increase (*p* = 0.175) was seen in practice, suggesting that behavioral changes may require more time to consolidate. Overall KAP score demonstrated a high effect size (Cohen’s *d* = 2.15, *p* ≤ 0.001) suggesting a preliminary association with KAP change in this uncontrolled pilot sample. All interventions resulted, however, in substantial distributional shifts in all the domains of KAP, as shown in Fig. 1, and a statistically significant increase in knowledge and attitude, while a shift in practice (although not statistically significant) was observed. Figure 1. Distribution of knowledge, attitude, and practice (KAP) categories pre- and post-intervention among older adults with NCDs. The most significant change was in knowledge with a significant decrease in the ‘insufficient’ level and a sharp increase at the ‘very satisfactory’ level, indicating better awareness and understanding. There was also positive attitude shift with a greater proportion of respondents shifting to the “sufficient” and “very satisfactory” categories reflecting improvement in attitude. Practice demonstrated a progressive change, but a greater number of participants in the higher categories suggests behavioral change, although it was not statistically significant. In general, the knowledge and attitude scores were found to have significantly improved while the practice score improved numerically but failed to attain statistical significance.

**Table 2:** Pre- and post-intervention changes in knowledge, attitude, practice, and overall KAP scores after the SMS intervention. Pre- and post-intervention scores were compared using paired-sample *t*-tests. Values are presented as mean ± SD. Mean differences were calculated as pre-intervention minus post-intervention scores; therefore, negative values indicate improvement after the intervention. Cohen’s *d* was used to estimate effect size. KAP, knowledge, attitude, and practice; CI, confidence interval; SMS, short message service.

Mean score	Pre/post	Mean ± SD	Mean difference	Std. deviation	Std. error mean	95% Confidence Interval of the difference	Cohen’s *d*	Sig. (two-tailed)
Knowledge	Pre	39.13 ± 9.59	−40.61	26.29	2.96	−46.50, −34.72	3.29	≤0.001
	Post	79.75 ± 14.56						
Attitude	Pre	35.29 ± 9.23	−24.43	17.43	1.96	−28.33, −20.53	1.77	≤0.001
	Post	59.72 ± 17.16						
Practice	Pre	27.63 ± 18.66	−3.58	23.29	2.62	−8.80, 1.63	0.18	0.175
	Post	31.22 ± 16.48						
Overall KAP	Pre	34.02 ± 7.56	−22.88	14.28	1.61	−26.07, −19.68	2.15	≤0.001
	Post	56.89 ± 13.00						

**Notes.**

Score of knowledge and attitude were ordinal Likert scales, whereas the practice scores consisted of binary and categorical variables of diet and physical activity. All the outcome variables were found to be normally distributed. Paired-sample *t*-tests were adopted to evaluate the differences between pre- and post-intervention. Cohen’s *d*: effect size (small ≥0.2, moderate ≥0.5, large ≥0.8). Mean differences represent post-intervention minus pre-intervention scores; negative values indicate that post-intervention scores were higher than pre-intervention scores, reflecting improvement.

### Regression analysis of factors influencing KAP scores

Table 3 shows that regression analysis indicates that the model statistically accounts for 97.3% of the variance in KAP (*R*^2^ = 0.973), but the result has to be taken with great caution, since it is based on a small number of pilots and these associations should be treated as exploratory and hypothesis generating, and not as confirmatory, until replicated at a larger sample size. The adjusted *R*^2^ value is 0.969 which suggests that 96.9% of the variance in KAP is explained after adjustment for the number of predictors, but this must be interpreted with caution due to the small sample size for the number of predictors. The standard error of estimate (SE) is 2.503, indicating how different the predicted values are from the actual values; these estimates should not be interpreted as more than a pilot study with a small sample size. Additionally, the results of an analysis of variance (ANOVA) show that the model is statistically significant in this sample (*F* = 274.325, *p* ≤ 0.001), which means that at least one of the independent variables in the model are predictive of KAP, but this needs to be replicated in a larger sample before conclusions can be drawn.

**Table 3:** Linear regression analysis of sociodemographic and lifestyle factors associated with change in overall KAP scores.

Model	Unstandardized coefficients		Standardized coefficients	Sig.	95.0% Confidence Interval for B	
	B	Std. error	Beta		Lower bound	Upper bound
Gender	−2.864	1.025	−0.067	0.007	−4.908	−0.819
Age	−0.985	0.098	−.646	≤0.001	−1.179	−0.790
BMI (kg/m^2^ )	0.003	0.061	0.001	0.957	−0.118	0.125
Marital status	2.234	1.176	0.082	0.062	−0.113	4.581
Education level	6.641	1.165	0.379	≤0.001	4.316	8.966
Work status	1.834	1.082	0.103	0.094	−0.324	3.992
Living status	4.263	1.933	0.150	0.031	0.406	8.120
Physical activity	−0.116	0.255	−0.009	0.650	−0.625	0.393
Smoking status	−0.348	0.332	−0.022	0.299	−1.011	0.315

**Notes.**

Independent variables included sociodemographic factors (age, gender, education level, marital status, work status, living status) and lifestyle-related predictors (BMI, physical activity level, smoking status). Reference categories: gender (female); education level (high school or less); work status (employed); living status (living with family); smoking status (current smoker); physical activity (not exercising).

B unstandardized coefficient Beta standardized coefficient CI confidence interval

*p* < 0.05 considered statistically significant.

In this exploratory model, age, education, gender, and living status were statistically significant factors associated with KAP improvement, but BMI, smoking, and physical activity were not. It should be noted that these associations should be viewed as hypothesis-generating and not as confirmed predictors of KAP improvement; these associations require replication in larger, independent samples before informing intervention design or policy. Age had a significant negative effect on KAP disparity (B = −0.985, *β* = −0.646, *p* ≤ 0.001). This indicates that younger participants showed greater improvement in KAP than older participants, with KAP disparity decreasing by approximately 0.985 units with each additional year of age. The 95% confidence interval (−1.179 to −0.790) confirms the strength of this effect. Education was the factor most strongly associated with KAP improvement (*B* = 6.641, *β* = 0.379, *p* ≤ 0.001). Participants with higher educational attainment experienced significantly greater improvement in KAP scores, with an average increase of 6.641 units. The confidence interval (CI) (4.316–8.966) supports the significance of this effect. Gender showed a statistically significant negative association with KAP disparity (B = −2.864, *β* = −0.067, *p* = 0.007). This indicates that male participants experienced less improvement in KAP than female participants, with a 2.864-unit decrease in KAP disparity for males. The confidence interval (CI) (−4.908 to −0.819) confirms the reliability of this result. Living status showed a statistically significant positive association with knowledge, skills, and practices (KAP) variation (*B* = 4.263, *β* = 0.150, *p* = 0.031). Participants living alone showed greater improvement in KAP scores than those living with families, with an increase of 4.263 units in KAP variation. The confidence interval (0.406–8.120) supports the reliability of this result.

## Discussion

To our knowledge, this pilot study is the first to evaluate the feasibility and preliminary effects of SMS-based NCD prevention messaging among older adults in Jordan and the Middle East and North Africa (MENA) region. After an intervention based on cultural adaptation and a 30-day period, preliminary improvements in NCD-related knowledge and attitudes were observed.

These improvements were larger than what we expected for knowledge scores (40.61 points; Cohen’s *d* = 3.29; *p* ≤ 0.001) and attitude scores (24.43 points; Cohen’s *d* = 1.77; *p* ≤ 0.001) after the brief time period of the intervention. In fact, this is an unexpectedly positive outcome; effect sizes this large are usually only achieved by engaging in meaningful behavior for 3–6 months. The notable gains in knowledge and attitudes may be attributed to this group’s receptiveness to culturally adapted, accessible health messages, and lend support to the rationale for proceeding to a longer, fully powered trial. The improvements in knowledge and attitude match theoretical models that propose a relationship between exposure to health information and readiness to change in both cognitive and motivational components of health behaviors; and with empirical studies demonstrating that presentation of health information that is accessible and culturally relevant can influence both the cognitive and motivational aspects of health behaviors (Heine et al., 2021; Hagger, 2025).

The total KAP scores increased significantly by 22.88 points (Cohen’s *d* = 2.15, *p* ≤ 0.001). The regression analysis showed that educational attainment was most strongly associated with KAP improvement (*β* = 0.379, *p* ≤ 0.001). Younger age (*β* = −0.646, *p* ≤ 0.001), female sex (*β* = −0.067, *p* = 0.007), and living alone (*β* = 0.150, *p* = 0.031) were also associated with greater KAP improvement. Against our hypothesis, lifestyle factors (BMI, smoking status, physical activity) were not found to be significantly associated with improvements in KAP, meaning that the demographic factors are more determinant to health education receptivity than the existing health behaviors. The results are consistent with previous studies indicating that an SMS-based approach has potential for managing NCDs, particularly among older adults with mobility challenges and limited access to traditional health education (Kampmeijer et al., 2016) but are not causal inferences about effectiveness.

The demographic and health characteristics of the study sample give a fundamental perspective for interpreting the findings of this study. The population of this cohort is predominantly middle-aged (mean age 57.33 years) and a BMI of 28.23 kg/m^2^ indicates that their population is at risk for numerous health complications associated with obesity and aging (Pollack et al., 2020). The high proportion of females (87.3%) indicates that any health outcomes observed may be affected by gender differences and gender specific health interventions are warranted (Hiller, Schatz & Drexler, 2017). Marital status revealed that most participants were married, which may influence social support systems and lead to improved health behaviors (Hiller, Schatz & Drexler, 2017). The high proportion of participants reporting no physical activity (57.0%) is concerning, particularly given the prevalence of hypertension and diabetes within this sample (CDC, 2024). Furthermore, 64.6% of the respondents indicated that they were currently smokers, creating a serious public health problem which might exacerbate the current health conditions (CDC, 2024). Overall, these demographic and health trends reflect the complexity of the health of this population and highlight the need for multifaceted approaches to meet the complex health needs of this population. As this study had no control group and used a convenience sample, attribution of causality is not possible and generalizability is limited. The large effect sizes observed for knowledge (Cohen’s *d* = 3.29) and attitude (Cohen’s *d* = 1.77) support proceeding to a fully powered randomized controlled trial.

Although gains in knowledge and attitudes were significant, changes in practice were modest and did not reach statistical significance, suggesting that more time is needed to consolidate behavioral change into routine (Hagger, 2025). The positive trend in the perception of the progression in practice scores is considered a preliminary step towards positive behavioral change and may require additional interventions to consolidate and embed such practices within routine. This aligns with the literature in question that discusses the fact that processes of behavioral change are complex, and can take time and continuous efforts (Yin et al., 2024). Furthermore, the Health Maintenance Consortium points out that behavior change is important, but maintaining change is difficult. Lasting behavior change is often achieved through the use of support systems and/or long-term interventions to help prevent a recurrence of previous behaviors or a “relapse” (Ory et al., 2010). The observed gains in knowledge and attitudes are encouraging, but future interventions should consider strategies that help in the transfer of this knowledge to effective and consistent intervention among targeted participants. These gains may be due to various mechanisms: daily message repetition may have facilitated information retention through spaced learning (Cepeda et al., 2006), the concise format of SMS messaging decreased the cognitive load among older people, and even culturally adjusted the content made in the message more relevant, as it is proposed in the Elaboration Likelihood Model. However, the gap between change in attitude and practice of behavior suggests that without studying environmental barriers and the habit of behavior process, knowledge is not sufficient without the need for extended intervention period (Lally et al., 2010). The exploratory regression analysis indicated that several sociodemographic variables were associated with KAP change. The pattern seen in this exploratory model suggested that age, education, gender, and living status are factors that may influence receptivity to educational intervention in this sample. Similar to past studies that highlighted how other personal characteristics influence health behaviors and comprehension and utilization of health-related knowledge (Hernandez, Margolis & Hummer, 2018; Jahan et al., 2020), these findings indicate that personal background traits have a strong influence on health behaviors and understanding and use of health-related knowledge. In the present study, education was the factor most strongly associated with KAP improvement. The improvements of KAP were found to be better among people with higher level education, as previous studies found a relationship between education and better health literacy and awareness of health promotion interventions. Age was also a dynamic variable, with younger people being more flexible and willing to learn, especially when information is provided on a digital or mobile platform. Hence, this highlights the importance of modifying and customizing intervention strategies to suit different age groups for better outcomes (Hernandez, Margolis & Hummer, 2018; Jeon et al., 2020). Unexpectedly, lifestyle-related factors, including body weight, smoking habits, and physical activity levels, did not have a significant impact on KAP outcomes. This indicates that, although lifestyle choices matter, demographic factors may have a more direct and significant effect on the initial involvement of an individual with health education and awareness.

Additionally, our findings are aligned with previous literature supporting the effectiveness of mobile-based education in health promotion. Studies have shown that SMS interventions may improve knowledge, attitudes, and health behaviors significantly across multiple populations. For instance, in Iran, Goodarzi et al. (2012) demonstrated significant improvements in diabetes self-management following a text-based educational program. Similarly, a review emphasized the behavioral impact of SMS in disease prevention and management, and the need to implement such successful tools for health-related changes. Furthermore, meta-analyses by other researchers further demonstrated the effectiveness of SMS as a scalable, cost-effective method to improve health knowledge and engagement, specifically among underserved or hard-to-reach populations (Head et al., 2013; Gelgelo et al., 2023). Based on these findings, our study suggests that delivering targeted educational messages *via* SMS to educate individuals about NCDs was feasible and had a positive impact on KAP outcomes. To the best of our knowledge, no systematic review of the topic on SMS interventions on NCD prevention in Arab older adults has been conducted, but wider reviews have indicated the efficacy of SMS as a health behavior change intervention (Head et al., 2013). This research is the first to provide a validated Arabic NCD-KAP measure and to suggest that theory-based, culturally sensitive SMS messaging is associated with large preliminary effect sizes in this low-resource setting, a finding that warrants confirmation through controlled study designs. The low cost of interventions indicates that it can be implemented on a national level *via* relationships between the Ministry of Health and mobile network providers, which has been observed in other regions (Mbuagbaw et al., 2015).

These findings suggest that the relationship between knowledge acquisition and behavioral change may be more complex than traditionally assumed and warrants further investigation. The lack of change in behavior despite the notable exposure to knowledge challenges the linear Knowledge–Attitude–Practice framework, indicating that structural factors can limit behavior even though it is motivated. The unexpected finding that living alone was associated with greater KAP improvement is not in line with expectations concerning the role of social support (Holt-Lunstad, Smith & Layton, 2010), which may indicate the existence of unmeasured confounding or special benefits of SMS among socially isolated people. Finally, the *R*^2^ value (0.973) is exceptionally high for behavioral research, raising the possibility of model overfitting given the small sample size relative to the number of predictors, and one needs to be very careful when interpreting the results. While statistically significant, this effect size is rarely seen in behavioral intervention research and is likely due, in part, to the small sample size (*n* = 79) that was used when compared to the number of predictors entered into the model. Therefore, all regression results presented in this study including the factors identified as predictors of changes in KAP must be considered exploratory and hypothesis generating and not confirmed or generalizable predictors for the purpose of informing intervention design or policy and should be replicated in larger, independent samples.

### Future research directions

To conclude on the effectiveness and based on these pilot results, we suggest the following: (1) a fully powered, randomized controlled trial with 6–12 month follow-up using two arms to satisfactorily determine effectiveness and maintenance of behavior change with the use of the effect sizes of the present pilot (Cohen *d* = 2.15 overall KAP) to determine the sample size; (2) qualitative studies to identify the barriers and facilitators of practice change in high and low responders; (3) component dismantling studies to determine the relative contributions of message repetition, cultural tailoring, and theoretical framing; (4) pragmatic effectiveness studies in the primary care system in Jordan to establish how effective it can be in comparison to traditional methods; and (5) testing combined interventions that pair SMS messaging with additional behavioural-support strategies. Evidence to inform regional policy recommendations would be strengthened through cross-cultural replication in other Arab countries.

## Strengths and Limitations

Several methodological strengths of this study increase its credibility and applicability. Using a culturally adapted and validated Arabic version of the CVD KAP29 questionnaire had improved the reliability and contextualization of the data, reflecting the participants’ CVD KAP. The intervention was a carefully grounded approach, appropriate for social cognitive theory and other previous systems of behavior change that were used in the field of behavioral health promotion. Furthermore, the single arm pre-post design and sample size (*n* = 79) was appropriate for this pilot study as it would be appropriate for determining feasibility and preliminary estimates of effects to be used in future definitive studies. High retention and adherence rates and minimized attrition bias throughout the study adds to the internal validity. The recruitment strategy (recruited from four geographically distinct primary care centers all over Amman) also added to the representativeness of the sample and reduced geographic and socioeconomic selection bias. The actual educational SMS messages’ language accessibility, cultural tailoring and international guidelines resulted in high participation and understanding. But there are limitations to the study, nevertheless. Because it was a pilot feasibility study, it was not a chance design decision that there was no randomized control group; this study was not designed to test for causality, but rather to see the feasibility of interventions and to get some idea of effect size to be used in future studies with larger and better powered samples. However, it cannot be excluded because of confounding effects and regression to mean. The *R*^2^ value is very high (0.973), and this is statistically significant, but it might be a result of model over-fitting. These preliminary regressions are to be replicated on larger and independent samples before these predictors can be established. One issue related to this conclusion is that the intervention duration was relatively short (one month), which may not have been long enough to produce a significant behavior change in the practice domain that takes more time for durable effects. Furthermore, while psychometric testing of the Arabic-adapted instrument was conducted within the current study sample, independent external validation in a separate population is still needed before the tool can be recommended for widespread application. Future validation studies should evaluate the factor structure using independent and larger samples through confirmatory factor analysis. Because psychometric evaluation and intervention testing were conducted within the same pilot sample, the measurement properties of the adapted instrument may be optimistic and should be interpreted accordingly. In addition, the lack of personalization to individuals’ needs or differential health literacy levels of participants, which these messages could achieve, was also an identified limitation of the SMS messages. Finally, the size of the sample was sufficient, but it was selected through convenience sampling, which may limit the extension of the results to the broader population of older adults with NCDs in Jordan.

## Conclusion

The pilot study shows that culturally modified SMS-based educational interventions are possible, acceptable, and exhibit encouraging initial outcomes in relation to knowledge and attitudes in the context of NCDs among Jordanian elderly patients. Results from the knowledge and attitude scores before and after the intervention suggest that mobile health (mHealth) interventions grounded in behavioral theory and tailored to the sociocultural context could be a promising health promotion approach with aging populations, pending confirmation in a controlled trial with comparison group to assess efficacy. Although there were minor behavioral practices changes, we observed a trend that shows that a long-term intervention might be needed to consolidate lifestyle shifts. The intervention engaged older adults to understand NCDs risk factors, preventive strategies, and healthy behaviours using evidence-informed message content derived from the Social Cognitive Theory and adapted from international frameworks.

The unidirectional SMS format appeared to be a viable, scalable, and potentially cost-effective approach to public health education for individuals with limited access to health services or low health literacy, subject to confirmation in a controlled study. The findings of this research support the extension of mHealth interventions for preventing NCDs across low- and middle-income countries. Such pilot results provide solid justification and preliminary data to support a fully powered randomized controlled trial, including longer follow-up times (6–12 months), a control comparison group, tailored messaging, and objective behavioral outcome measures to provide conclusive information on intervention effectiveness and sustenance. These findings should be confirmed through adequately powered randomized controlled studies with longer follow-up periods before broader implementation recommendations can be made.

##  Supplemental Information

10.7717/peerj.21643/supp-1 Supplemental Information 1 Anonymized SPSS dataset used in the analysis

10.7717/peerj.21643/supp-2 Supplemental Information 2 Highlighted box

10.7717/peerj.21643/supp-3 Supplemental Information 3 STROBE Checklist

## Additional Information and Declarations

### Data Availability

The following information was supplied regarding data availability: The data is available in the Supplemental Files.
