# Supplementary material for: Feasibility and preliminary effects of a culturally adapted short message service intervention for non-communicable disease prevention in older adults: a pilot study
Source: PeerJ. 2026 Aug 24;14:e21643. doi: 10.7717/peerj.21643 (PMC13525760; doi:10.7717/peerj.21643)
Supplement: Supplementary file 1 [file peerj-14-21643-s003.pdf]

STROBE Statement—Checklist of items that should be included in reports of ***cohort studies***

| | Item No | Recommendation |
|---|---|---|
| **Title and abstract** | 1 | **Title**: "pilot study" **Abstract**: "one-group pre-post design" **page 1** |
| | | **Abstract**: Complete structured abstract **page 1** |
| **Introduction** | | |
| Background/rationale | 2 | **Introduction**: Paragraphs 1-5 (NCD burden, digital health, gaps) **page 2-4** |
| Objectives | 3 | **Introduction**: Final paragraph (3 objectives listed) **page 4** |
| **Methods** | | |
| Study design | 4 | Materials and methods - Study design **page 4-5** |
| Setting | 5 | **Participants**: "four governmental primary healthcare centers in Amman, Jordan, April-June 2024". **page 5** |
| Participants | 6 | **Participants**: 5 inclusion + 4 exclusion criteria . **page 5** |
| | | (*b*) **N/A** - Not a matched study |
| Variables | 7 | **Study questionnaire**: Knowledge (12), Attitude (10), Practice (7 items) **page 7-8** |
| Data sources/ measurement | 8* | **Study questionnaire** + **Psychometric analysis page 7-9** |
| Bias | 9 | **Participants**: screening checklist **Study questionnaire**: standardized instructions **page 5, 8** |
| Study size | 10 | **Sample size**: G*Power, Cohen's d=0.5, n=45 minimum **page 6** |
| Quantitative variables | 11 | **Statistical analysis**: KAP 5 categories, normality testing **page 9-10** |
| Statistical methods | 12 | **Statistical analysis**: Paired t-test, Cohen's d, regression **page 9-10** |
| | | **Statistical analysis**: Regression with sociodemographic factors **page 10** |
| | | **Statistical analysis**: "listwise deletion" **Sample size**: per protocol **page 6, 10** |
| | | **Sample size**: "21 participants withdrew/lost" **page 6** |
| | | **Statistical analysis**: Model assumptions, residuals checked **page 10** |
| **Results** | | |
| Participants | 13* | **Feasibility**: 120 approached → 100 enrolled → 79 completed **page 10** |
| | | Sample size: withdrew, lost to follow-up, incomplete **page 6** |
| | | NOT INCLUDED |
| Descriptive data | 14* | Demographic and Health Characteristics section **page 11** |
| | | NOT EXPLICITLY STATED (n=79 throughout) |
| | | Materials and methods - Study design: "text messages daily over 4 weeks" Page 5 Materials and methods - Intervention procedure: "delivered for 25 consecutive days" Page 6 Materials and methods - Study questionnaire: "baseline (pre-intervention) and post-intervention (after receiving 30 text messages over one month)"Page 8 Results - Feasibility and Acceptability Outcomes: "79 participants filled out the baseline and follow-up assessment" Page 10 |

| | | |
|---|---|---|
| Outcome data | 15* | **Pre-Post Changes in KAP**, **Table 1**, **Figure 1 page 11-12** |
| Main results | 16 | **Pre-Post Changes**: t-test<br>**Regression**: Adjusted estimates **page 11-12**<br>**Statistical analysis**: ≤20, 21-40, 41-60, 61-80, >**80 page 10, 12**<br>NOT APPLICABLE |
| Other analyses | 17 | **Regression Analysis**: Predictors, model diagnostics **page 12** |
| **Discussion** | | |
| Key results | 18 | **Discussion**: Paragraphs 1-2 (Knowledge +40.61, d=3.29; Attitude +24.43, d=1.77) page 12-13 |
| Limitations | 19 | **Strengths and Limitations**: No control, convenience sample, short duration **page 17-18** |
| Interpretation | 20 | **Discussion** + **Conclusion page 12-18** |
| Generalisability | 21 | **Strengths and Limitations**: Convenience sampling limits extension **page 18** |
| **Other information** | | |
| Funding | 22 | **Declaration - Funding**: "This study was not funded" **page 19** |

University of Petra Ethics (E/H/4/10/2024), Informed Consent Statement, Declaration: "No conflicts", Data Availability Statement, Author Contributions **page 19**

*Give information separately for exposed and unexposed groups.

Study Flow Diagram

┌─────────────────────────────────┐ │ Assessed for eligibility │
│ (n = 120) │ └─────────────┬─────────────────┘ │ ▼
┌─────────────────────────────────┐ │ Enrolled and provided
consent │ │ (n = 100) │ │ Recruitment rate: 83.3% │
└─────────────┬─────────────────┘ │ ▼
┌─────────────────────────────────┐ │ Completed baseline
assessment │ │ (n = 100) │ └─────────────┬─────────────────┘ │
▼ ┌─────────────────────────────────┐ │ Received 30-day SMS
intervention │ │ (n = 100) │ └─────────────┬─────────────────┘
│ ▼ ┌─────────────────────────────────┐ │ Completed follow-up
assessment │ │ (n = 79) │ │ Retention rate: 79% │
└─────────────┬─────────────────┘ │ ▼
┌─────────────────────────────────┐ │ Included in final analysis │
│ (n = 79) │ └─────────────────────────────────┘ Lost to follow-
up (n = 21):
