# Supplementary material for: Feasibility and preliminary effects of a culturally adapted short message service intervention for non-communicable disease prevention in older adults: a pilot study
Source: PeerJ. 2026 Aug 24;14:e21643. doi: 10.7717/peerj.21643 (PMC13525760; doi:10.7717/peerj.21643)
Supplement: Supplementary file 2 [file peerj-14-21643-s002.docx]

1. SMS Intervention has an Effective Impact on the NCD Knowledge and Attitudes. One-month SMS intervention that had been culturally based, providing 30 theory-based health messages, was found to have significant improvement on knowledge (Cohen’s d=3.29) and attitudes (Cohen’s d=1.77) about NCD prevention among older adults in Jordan. This proves that mobile messaging is a cost-effective and scalable way of improving health literacy in resource-constrained environments.

2. Intervention is predicted by education and age. The best predictors of improvement in knowledge were higher educational attainment and younger age. Gender and living alone (female gender) were also significantly associated. These results indicate that the demographically specific interventions should be developed especially focusing on less educated older adults by providing them with simplified messaging methods.

3. Change in behavior takes a long period of involvement. Although the knowledge and attitudes were enhanced greatly, practice changes were not high after one month. Awareness to behavioural change takes a longer period of intervention, repeated reinforcement, and in some cases, a combination of digital messages with physical support to entrench healthy lifestyle practices.

4. First Arabic mHealth NCD Prevention Evidence. The proposed research will give a ground breaking evidence on the use of culturally adapted Arabic SMS interventions to reach older adults with NCDs in Jordan. The low cost and availability of text messaging make this method useful to the Middle East countries that have increasing burdens of NCDs and limited healthcare capabilities.
